# What racial disparities exist in the prevalence of perinatal bipolar disorder in California?

**DOI:** 10.3389/fpsyt.2025.1550634

**Published:** 2025-05-16

**Authors:** Mercy Eigbike, Rebecca J. Baer, Nichole Nidey, Nancy Byatt, Xavier R. Ramirez, Hsiang Huang, Crystal T. Clark, Avareena Schools-Cropper, Scott P. Oltman, Laura L. Jelliffe-Pawlowski, Kelli K. Ryckman, Karen M. Tabb

**Affiliations:** ^1^ Department of Psychiatry and Psychology, Carle Foundation Hospital, Urbana, IL, United States; ^2^ Carle Illinois College of Medicine, Urbana, IL, United States; ^3^ Department of Obstetrics, Gynecology, & Reproductive Health Sciences, School of Medicine, University of California, San Francisco, San Francisco, CA, United States; ^4^ UCSF California Preterm Birth Initiative, University of California, San Francisco, San Francisco, CA, United States; ^5^ Department of Pediatrics, School of Medicine, University of California, San Diego, San Diego, CA, United States; ^6^ College of Public Health, University of Iowa, Iowa, IA, United States; ^7^ Department of Psychiatry and Behavioral Sciences, UMass Chan Medical School, Shrewsbury, MA, United States; ^8^ School of Social Work, University of Illinois at Urbana-Champaign, Urbana, IL, United States; ^9^ Department of Psychiatry, Cambridge Health Alliance, Cambridge, MA, United States; ^10^ Department of Psychiatry, University of Toronto, Toronto, ON, Canada; ^11^ Centers for Medicare and Medicaid Services, Office of Minority Health, Baltimore, MD, United States; ^12^ Department of Epidemiology & Biostatistics, School of Medicine, University of California, San Francisco, San Francisco, CA, United States; ^13^ Institute of Global Health Sciences, University of California, San Francisco, San Francisco, CA, United States; ^14^ Rory Meyers College of Nursing, New York University, New York, NY, United States; ^15^ Department of Epidemiology and Biostatistics, School of Public Health-Bloomington, Indiana University, Bloomington, IN, United States; ^16^ Beckman Institute for Advanced Science and Technology, University of Illinois, Urbana-Champaign, IL, United States

**Keywords:** bipolar disorder, perinatal, minority women, multiracial, mental health

## Abstract

**Purpose:**

Mental health conditions are the leading cause of preventable maternal mortality and morbidity, yet few investigations have examined perinatal bipolar disorders. This study sought to examine racial differences in the odds of having a bipolar disorder diagnosis in perinatal women across self-reported racial groups in a large sample in California, USA.

**Method:**

This cross-sectional study uses data from 3,831,593 women who had singleton live births in California, USA from 2011 to 2019 existing in a linked dataset which included hospital discharge records and birth certificates. International Classification of Diseases codes were used to identify women with a bipolar disorder diagnosis code on the hospital discharge record. Medical charts and birth certificate data was used to extract information on clinical and demographic covariate characteristics. Multivariable logistic regression was used to estimate the odds of having a bipolar disorder diagnosis across different self-reported racial groups.

**Results:**

We identified 19,262 women with bipolar disorder diagnoses. Differences in the presence of a bipolar disorder diagnosis emerged by self-reported race. In the fully adjusted model, Multiracial (selection of two races self-reported) women, compared to single-race White women had the highest odds of having a bipolar disorder diagnosis. Further examination of the all-inclusive Multiracial category revealed differences across subgroups where White/Black, White/American Indian Alaskan Native, and Black/American Indian Alaskan Native women had increased odds for bipolar disorder compared to single race White women.

**Conclusions:**

Differences in bipolar disorder diagnoses exist across racial categories and when compared to White women, Multiracial women had the highest odds of bipolar disorder and thus represent a perinatal population of focus for future intervention studies. The increased burden of mental health problems among Multiracial women is consistent with recent research that employs disaggregated race data. More studies of Multiracial women are needed to determine how self-reported racial categories are related to increased risk for perinatal bipolar disorder.

## 1 Introduction

Mental health conditions, including bipolar disorder, are leading preventable causes of maternal mortality and morbidity (1). Bipolar disorder has an estimated lifetime prevalence of 1%–2.4% and has a similar prevalence in men and women. Bipolar disorder is associated with functional impairment, disability, and increased risk for suicide (2–4). Premature mortality can also occur with bipolar disorder due to associated chronic medical conditions such as diabetes and cardiovascular disease (5). Given the severe risks associated with bipolar disorder and the increase of maternal complications there is a need to examine the prevalence of bipolar disorder across the life course.

The perinatal period (during pregnancy and up to one year postpartum) is a time during which there is an increased risk for the development and recurrence of mood disorders (6, 7). Bipolar disorder episodes during pregnancy are associated with risks for adverse perinatal outcomes including neonatal morbidities, congenital malformations, and neonatal hospital readmissions (8). In addition, studies report 5%–22.6% positive screening rates for bipolar disorder in the perinatal period (9–12). Within these previous studies on bipolar disorders, there remains a need to document patterns across the population and any disparities.

Previous studies report significant racial/ethnic differences in the diagnosis of mental health disorders in the United States, with most minority groups having lower rates of psychiatric diagnosis including depression (13). Other studies, on the contrary, report a greater risk of serious mental illness, including bipolar disorder, among minority adults (14–16). However, very few of these studies focus on the perinatal period and examine the risk among samples of racially diverse women. Accordingly, the aim of this study is to examine the odds of bipolar disorder across racial groups, including women self-reporting as a single race and Multiracial (two or more races), in a perinatal population residing in California, U.S.A.

## 2 Materials and methods

**Figure 1:**
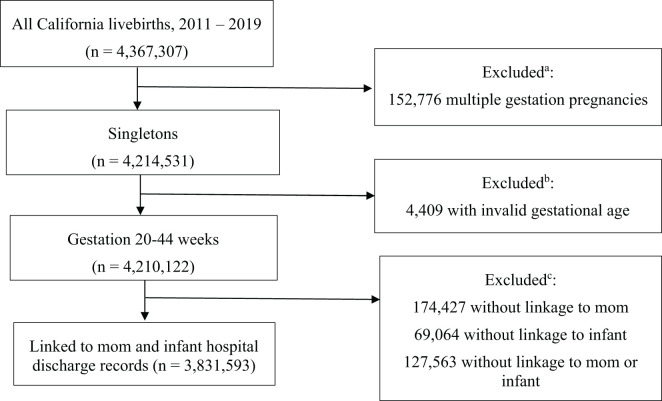
Sample selection ^a^Because multiple gestation was excluded, it should be noted that findings in this paper may not be applicable to multiple gestation pregnancies ^b^Because invalid gestational age was excluded, some of the population is reduced. Records without valid gestational age have not been extensively examined, however it is possible that the records excluded are biased by race, mental health diagnoses, and other factors ^c^Bipolar diagnosis and other diagnoses required linkage to hospital discharge records. However, we have demonstrated that a higher percentage of those not linked are BIPOC (PMID: 39625748) implying a limitation to our findings.

The study population for this cross-sectional study included women who had singleton live births in California from 2011-2019 (N=3,831,593). This cohort has been previously described in prior studies examining perinatal mental health disorders (17, 18). Hospital discharge data, including diagnosis and procedure codes based on the International Classification of Diseases, Ninth Revision, Clinical Modification (ICD-9-CM) (19) were linked to vital records (birth certificate and death certificate) (see **Figure 1**). Detailed information on maternal characteristics recorded up to one year prior to and after delivery maintained by the California Department of Health Care Access and Information.

### 2.1 Measures

The main outcome of this study, a bipolar disorder diagnosis, was based on the following ICD-9-CM codes: 296.0, bipolar I disorder, single manic episode; 296.1, manic disorder, recurrent episode; 296.4, bipolar I disorder, most recent episode (or current) manic; 296.5, bipolar I disorder, most recent episode (or current) depressed; 296.6, bipolar I disorder, most recent episode (or current) mixed; 296.7, bipolar I disorder, most recent episode (or current) unspecified; 296.8, bipolar disorder, unspecified; 296.89, bipolar II disorder; 296.9, other and unspecified episodic mood disorder or ICD-10 codes F30 or F31. Women with any of these ICD codes reported in their hospital discharge records were considered to be positive cases of bipolar disorder and women without any of these codes were considered controls. Specifically, these positive cases represent the proportion of clinically documented bipolar disorder diagnoses in hospital discharge records.

Information on race and ethnicity, the main exposures of interest for this study, were obtained from maternal self-report on the birth certificate records. We used self-reported race categorized as White, Black, Asian, American Indian/Alaskan Native (AIAN), Hawaiian Pacific Islander (HAWPI), Other race, and Multiracial (defined as two or more races). Multiracial women were subcategorized into White/Black, White/Asian, White/AIAN, White/HAWPI, White/Other, Black/Asian, Black/AIAN, Black/HAWPI, Black/Other, HAWPI/Asian, and HAWPI/Other. Similarly, ethnicity was self-reported as Hispanic/Latino/a and Not Hispanic/Latino/a.

Clinical and demographic characteristics include age, education, insurance, the Special Supplemental Nutrition Program for Women, Infants, and Children (WIC) enrollment, medical conditions, substance use disorder, and psychiatric conditions. Birth certificate data including maternal age at delivery (18, 18–34 and >34); maternal education (<12 years, 12 years, >12 years); insurance type (private, Medi-Cal, self-pay, other); enrollment into WIC and parity (nulliparous or multiparous) was used for this study as predictors. ICD codes from hospital discharge data were used to identify individuals with the following diagnoses: gestational diabetes; gestational hypertension; substance use (alcohol, cocaine, cannabis, amphetamines and other psychostimulant, sedative, hypnotic, or anxiolytic, hallucinogen, opioid, smoking); anxiety; depression; and schizophrenia (**Supplementary Table 1**).

### 2.2 Statistical analysis

Maternal characteristics by bipolar disorder were compared using chi-square tests. The odds of having bipolar disorder by race and ethnicity were examined with logistic regression models. We estimated the odds of having bipolar disorder by self-reported race and ethnicity categories, where individuals who reported more than one race were combined into one group defined as Multiracial. The regression analyses were adjusted for maternal characteristics, including age at delivery, education, insurance type, and enrolment in WIC. In the fully adjusted model, we further adjusted for health conditions (gestational diabetes, gestational hypertension, depression, anxiety, schizophrenia, and substance use [alcohol, cocaine, cannabis, amphetamines and other psychostimulant, sedative, hypnotic, or anxiolytic, hallucinogen, opioid, smoking]). Lastly, because the Multiracial category combined heterogeneous groups, we examined Multiracial subgroups (combinations) within the inclusive Multiracial category with adjustments for the other covariates in a *post-hoc* analysis and share a supplementary table. Analyses were performed using software (SAS, Version 9.4; SAS Institute Inc, Cary, NC).

## 3 Results

The frequency distribution of maternal characteristics by bipolar disorder (yes/no) are presented in **Table 1**. In this sample of 3,831,593, a total of 19,262 people had bipolar disorder. Significant differences across maternal characteristics were present for all categories between those with bipolar disorder and those without bipolar disorder. **Table 2** presents the adjusted risks for bipolar disorder. Overall, race/ethnicity emerged as an important predictor for having a bipolar disorder diagnosis. In the regression analyses non-White single race racial minority categories of Black, Asian, and Native Hawaiian women were less likely to have a bipolar disorder diagnosis (OR:.94, CI.89-.99; OR:0.22, CI.22-.24 and OR:.38, CI.28-.51 respectively). For the Multiracial group there was an increased risk (OR: 1.67, CI 1.59, 1.76) and this group had the highest odds of bipolar disorder when compared to White women. The Multiracial category as a heterogenous group contained significant differences across Multiracial subgroups where White/Black and White/American Indian/Alaskan Native presented the greatest odds for bipolar disorder (see **Supplementary Table 2**). In a fully adjusted model salient socioeconomic factors, such as high school education or enrolled in WIC was associated with the odds of bipolar disorder.

**Table 1:** Maternal characteristics of women with and without bipolar disorder diagnostic codes.

Variables	Total	With bipolar disorder	Without bipolar disorder	*P*-value
	n (%)	n (%)	n (%)	
**Sample**	**3,831,593**	**19,262**	**3,812,331**	
**Race**				***< 0.0001***
White	2,689,314 (70.2)	13,458 (69.9)	2,675,856 (70.2)	
Black	193,729 (5.1)	2,273 (11.8)	191,456 (5.0)	
Asian	566,430 (14.8)	581 (3.0)	565,849 (14.8)	
American Indian/Alaska Native	17,475 (0.5)	244 (1.3)	17,231 (0.5)	
Native Hawaiian/Pacific Islander	15,286 (0.4)	49 (0.3)	15,237 (0.4)	
Other	16,689 (3.1)	203 (1.1)	116,486 (3.1)	
Multiracial	139,304 (3.6)	1,863 (9.7)	137,441 (3.6)	
Unknown	93,366 (2.4)	591 (3.1)	92,775 (2.4)	
**Hispanic/Latino**	1,865,469 (48.7)	5,523 (28.7)	1,869,946 (48.8)	***< 0.0001***
**Age at delivery (years)**				***< 0.0001***
< 18	58,755 (1.5)	339 (1.8)	58,416 (1.5)	
18 – 34	2,966,160 (77.4)	15,531 (80.6)	2,950,629 (77.4)	
>34	806,559 (21.1)	3,392 (17.6)	803,167 (21.1)	
Missing	119 (0.0)	*	119 (0.0)	
**Education (years)**				***< 0.0001***
< 12	621,631 (16.2)	3,523 (18.3)	618,108 (16.2)	
12	933,688 (24.4)	5,801 (30.1)	927,887 (24.3)	
>12	2,104,304 (54.9)	8,896 (46.2)	2,095,408 (55.0)	
Missing	171,970 (4.5)	1,042 (5.4)	170,928 (4.5)	
**Payer for delivery**				***< 0.0001***
Private	1,839,889 (48.0)	6,728 (34.9)	1,844,161 (48.1)	
Medi-Cal	1,741,899 (45.5)	11,504 (59.7)	1,730,395 (45.4)	
Self-pay	123,924 (3.2)	165 (0.9)	123,759 (3.3)	
Other	125,881 (3.3)	865 (4.5)	125,016 (3.3)	
**WIC**	**1,878,677 (49.0)**	**11,670 (60.6)**	**1,867,007 (49.0)**	***< 0.0001***
**Nulliparous**	1,475,537 (38.5)	7,629 (39.6)	1,467,908 (38.5)	***0.0017***
**Drug Use**	79,696 (2.1)	5,085 (26.4)	74,611 (2.0)	***< 0.0001***
**Alcohol Use**	7,541 (0.2)	877 (4.6)	6,664 (0.2)	***< 0.0001***
**Smoking History**	113,260 (3.0)	5,751 (29.9)	107,509 (2.8)	***< 0.0001***
**Gestational Diabetes**	409,786 (10.7)	2,300 (11.9)	407,486 (10.7)	***< 0.0001***
**Gestational Hypertension**	272,569 (7.1)	2,154 (11.2)	270,415 (7.1)	***< 0.0001***
**Anxiety**	101,327 (2.6)	4,893 (25.4)	96,434 (2.5)	***< 0.0001***
**Schizophrenia**	3,104 (0.1)	1,344 (7.0)	1,760 (0.1)	***< 0.0001***
Puerperal psychosis	90 (0.0)	9 (0.1)	90 (0.0)	*< 0.0001*
Depression	84,450 (2.2)	2,393 (12.4)	82,057 (2.2)	*< 0.0001*

*not displayed when n < 5.

**Table 2:** Adjusted logistic regression of bipolar disorder in perinatal women in the State of California.

Variables	Adjusted OR 95% CI	p-value
Race		
Black	0.94 (0.89, 0.99)	0.0134
Asian	0.22 (0.20, 0.24)	< 0.0001
American Indian/Alaska Native	1.14 (0.99, 1.31)	0.0683
Native Hawaiian/Pacific Islander	0.38 (0.28, 0.51)	< 0.0001
Other	0.58 (0.50, 0.67)	< 0.0001
Multiracial	1.67 (1.59, 1.76)	< 0.0001
Unknown	0.72 (0.66, 0.79)	< 0.0001
Hispanic/Latino	0.37 (0.36, 0.39)	< 0.0001
Age at delivery (years)		
< 18	1.21 (1.08, 1.36)	0.0014
>34	0.97 (0.93, 1.01)	0.0968
Education (years)		
< 12	0.99 (0.95, 1.04)	0.7721
>12	0.93 (0.90, 0.97)	0.0002
Payer for delivery		
Medi-Cal	1.23 (1.18, 1.28)	< 0.0001
Self-pay	0.58 (0.49, 0.68)	< 0.0001
Other	1.35 (1.25, 1.45)	< 0.0001
WIC	1.52 (1.46, 1.58)	< 0.0001
Nulliparous	1.11 (1.07, 1.15)	< 0.0001
Drug Use	3.87 (3.70, 4.04)	< 0.0001
Alcohol Use	2.68 (2.45, 2.94)	< 0.0001
Smoking History	3.54 (3.40, 3.69)	< 0.0001
Gestational Diabetes	1.30 (1.24, 1.36)	< 0.0001
Gestational Hypertension	1.28 (1.22, 1.34)	< 0.0001
Anxiety	6.59 (6.33, 6.86)	< 0.0001
Schizophrenia	33.31 (30.36, 36.54)	< 0.0001
Puerperal psychosis	4.01 (1.48, 10.89)	0.0064
Depression	1.07 (1.02, 1.13)	0.0133

## 4 Discussion

In this cross-sectional study of perinatal women in the State of California, the odds of having a bipolar disorder diagnosis varied by race and ethnicity. Overall, the proportion of bipolar disorder was 0.05% (n = 19,262). This finding is much lower than in past studies of the perinatal population and some previous studies reported the pooled prevalence of 2% for bipolar disorders (6, 12, 20). This difference may be due to a variety of reasons that warrant need for future investigations. Given what is known in existing evidence, our lower proportion of bipolar disorder could be due to a number of factors outside of the study such as lack of documentation, lack of detection, inaccurate documentation, or even misdiagnosis. In one study by Masters and colleagues including 574 postpartum women recruited from obstetrics clinics, 18.8% screened positive (using the Mood Disorder Questionnaire (MDQ)) for probable bipolar disorder, of which 20.8% were Black/African American, 64.6% were White, 4.2% were Asian, 8.3% were Multiracial, and 2.1% reported Other race (12). Single-race minority women, Black, Asian, Hawaiian/Pacific Islander, and Hispanic/Latine women had lower odds of probable bipolar disorder when compared to White women, and Asian women had the lowest odds. Although race/ethnicity was not the focus in the Masters et al. study, their results showed some similarity to ours, including lowest bipolar disorder positive screens in Asian women.

Our study finding of reduced odds of having bipolar disorder diagnosis among Black, Asian, and HAWPI and Hispanic/Latine women when compared to White women supports earlier observations suggesting lower diagnosis in these minority groups for reasons including misdiagnosis and unconscious bias (21–23). Contrary to our findings, Masters et al. had higher bipolar disorder positive screens (probable bipolar disorder) among Black women, with the lowest positive screens reported among Multiracial women. Masters and colleagues screened individuals who were recruited from 14 obstetric clinics compared to our population-based sampling framework, their Multiracial category included one or more races, and their results had data for Black as all-inclusive without any further subcategories. Furthermore, the low rate of bipolar disorder in our population-based study is likely a reflection of how we are ascertaining bipolar disorder. This study identified bipolar disorder by the presence of an ICD-9 diagnostic code on the hospital discharge records for women who have a linked birth certificate record. Not all women with bipolar disorder would necessarily have the diagnosis code reflected on their hospital discharge record, which suggests there is an underreporting of this condition and is a limitation of our study. Additionally, there exists the risk of misclassification of women with bipolar disorder as having unipolar depression (i.e. major depressive disorder).

As a novel contribution, our sample was drawn from California, which has a uniquely diverse population and included many women with multiple racial combinations. Our findings showed the greatest odds of bipolar disorder among Multiracial women. Among the different racial combinations self-reported by Multiracial women, bipolar disorder was highest among Black/AIAN women, followed by White/Black women and White/AIAN women. A previous study by Coleman et al. noted increased mental illness diagnoses among AIAN women compared to other minority races and non-Hispanic White women (13). The explanation as to why Multiracial AIAN and Multiracial Black women experience an increased risk is critical to uncover. There are many reasons including lack of access to quality healthcare, adverse social determinants of health and systemic racism. Like our findings for bipolar disorder, Coleman and colleagues noted the reduced risk of any psychiatric diagnosis for Asian women (13). This may be consistent with the negative perception of psychiatric illness in Asian women. More research is needed on specific and often underreported racial minority groups such as Asian and Multiracial women to document the patterns and characteristics of serious mental illness during the perinatal period.

Racial differences in bipolar disorder persisted among women in our cohort with bipolar disorder after adjusting for illicit drug use, obesity, adverse birth outcomes, gestational diabetes, gestational hypertension, and smoking. This is consistent with previous studies (20, 24–26) that showed more adverse pregnancy outcomes like gestational hypertension in women with bipolar disorder with increased risk of mood disorders in the postnatal period. Possible explanations for our finding of increased odds of medical comorbidity in women with bipolar disorder compared to those without bipolar disorder could be disease severity, limitations in self-care, and unknown substance abuse. These factors have been highlighted as important factors in other studies (27). Another factor that may be influential is the use of antipsychotics for treating bipolar disorder, many of which predispose women to developing metabolic syndrome (28). Although we were not able to investigate this relationship, examination in future studies will be critical.

The strengths of this study include the very large sample size and the diversity of residents of California, which allowed for examination of an extensive set of racial categories including some Multiracial groups. Despite the study strengths, this study has some limitations. First, the data captures information for women who have a diagnosis of bipolar disorder entered into their hospital discharge records, which may only include more severe clinical cases. It is also likely that the proportion of bipolar disorder in the single race minority groups studied is underestimated, as earlier studies have shown underutilization of mental health services by ethnic minority mothers (29). It is also possible that some individuals with bipolar disorder were misclassified in our sample. We found that 8.89% of women with a bipolar disorder diagnosis also had a diagnosis of schizophrenia. This overlapping of the presence of ICD-9 codes for two distinct psychiatric and mutually exclusive conditions is not surprising because ICD-9 documentation may not accurately capture the true psychiatric diagnosis (30). It will be important that future investigations into these patterns include the ability to conduct intensive chart review to examine documentation of diagnosis beyond ICD-9 codes.

Perinatal women are a group that has unmet mental health needs including those with bipolar disorder (9). With support from psychiatric specialists, including through consultation, resource and referrals, obstetrics settings can be an opportunity for identifying women with untreated bipolar disorder and unmet mental health needs (31, 32). For example, screening for depression could be expanded to include screening for bipolar disorder (33). We also need to better understand disparities existing in the diagnosis, prevalence, course, and treatment of bipolar disorder and compare to unipolar depression which may be different in minority women. Accordingly, future research is needed to develop best practices for identifying perinatal women in need of treatment for bipolar disorder and culturally responsive approaches for Multiracial minority women.

## 5 Conclusion

The increased odds of bipolar disorder among Multiracial women compared to White women in the perinatal period makes them an important group for increased intervention and prevention efforts. Steps should be taken to address these concerns proactively. This could include identifying Multiracial women and making sure to screen carefully for bipolar disorder especially if they screen positive for depression during the perinatal period. Considerations are needed when examining Multiracial women as a homogenous group as our findings revealed distinct patterns in the risk for bipolar disorder across Multiracial subgroups. Additionally, providers should be racially and culturally responsive when making diagnoses and serving perinatal women, especially those of minoritized racial groups. Health literacy measures should be put in place to ensure increased awareness by the public and providers concerning bipolar disorder among racial and ethnic minorities to address possible gaps in care.

## Data Availability

The data that support the findings of this study are available from the California Department of Health Care Access and Information (https://hcai.ca.gov/data-and-reports/request-data/) and California Vital Statistics (https://www.cdph.ca.gov/Programs/CHSI/Pages/Data-Applications.aspx), but restrictions apply to the availability of these data, which were used under California Committee for the Protection of Human Subjects IRB for the current study, and so are not publicly available.

## Supplementary material

Supplementary Table 1 Diagnostic Codes Used as Covariates.

Supplementary Table 2 Expansion of Multiracial groups and fully adjusted logistic regression results for specific Multiracial subgroups.
