# Supplementary material for: What racial disparities exist in the prevalence of perinatal bipolar disorder in California?
Source: Front Psychiatry. 2025 May 16;16:1550634. doi: 10.3389/fpsyt.2025.1550634 (PMC12288092; doi:10.3389/fpsyt.2025.1550634)
Supplement: Supplementary Table 1 — Diagnostic Codes Used as Covariates. [file Table1.docx]

**Supplemental Table 1: Diagnostic Codes Used as Covariates**

| ICD-9 diagnostic code | ICD-10 diagnostic code | Birth certificate indication | Diagnosis |
| --- | --- | --- | --- |
| 648.8 | P70.0, O24.4 | X | Gestational diabetes |
| 642.3 | O13 | X | Gestational hypertension |
| 303, 305.0 | F10 |  | Alcohol use |
| 304.2 | F11, F12, F13, F14, F15, F16, F18, F19 |  | Drug use |
| 300 | F41 |  | Anxiety |
| 293.89 | F53.1 |  | Puerperal psychosis |
| 295.7 | F20 |  | Schizophrenia |
| 296.2, 296.3, 311 | F32, F33, F34, F38, F39 |  | Depression |

**Supplemental Table 2. Expansion of Multiracial groups and fully adjusted logistic regression results for specific Multiracial subgroups**

|  | **Total** | **With bipolar disorder** | **Without bipolar disorder** | **Adjusted OR**  **95% CI** | **p-value** |
| --- | --- | --- | --- | --- | --- |
| White/Black | 33,311 (0.9) | 667 (3.5) | 32,644 (0.9) | 2.03 (1.86, 2.21) | < 0.0001 |
| White/Asian | 38,230 (1.0) | 276 (1.4) | 37,954 (1.0) | 1.18 (1.05, 1.34) | 0.0078 |
| White/American Indian/Alaska Native | 24,502 (0.6) | 541 (2.8) | 23,961 (0.6) | 2.16 (1.96, 2.37) | < 0.0001 |
| White/Native Hawaiian/Pacific Islander | 5,922 (0.2) | 48 (0.3) | 5,874 (0.2) | 1.08 (0.80, 1.44) | 0.6297 |
| White/Other | 20,747 (0.5) | 89 (0.5) | 20,658 (0.5) | 1.20 (0.97, 1.50) | 0.0951 |
| Black /Asian | 4,021 (0.1) | 45 (0.2) | 3,976 (0.1) | 1.04 (0.76, 1.43) | 0.7861 |
| Black /American Indian/Alaska Native | 3,935 (0.1) | 110 (0.6) | 3,825 (0.1) | 1.93 (1.56, 2.39) | < 0.0001 |
| Black /Native Hawaiian/Pacific Islander | 1,085 (0.0) | 19 (0.1) | 1,066 (0.0) | 1.61 (0.99, 2.63) | 0.0559 |
| Black /Other | 1,701 (0.0) | 25 (0.1) | 1,676 (0.0) | 1.62 (1.05, 2.49) | 0.0285 |
| Asian /American Indian/Alaska Native | 897 (0.0) | 10 (0.1) | 887 (0.0) | 1.24 (0.63, 2.42) | 0.5337 |
| Asian /Native Hawaiian/Pacific Islander | 3,201 (0.1) | 15 (0.1) | 3,186 (0.1) | 0.70 (0.42, 1.16) | 0.1669 |
| Other Multiracial | 1,752 (0.1) | 18 (0.01) | 1,734 (0.1) | 1.32 (0.79, 2.22) | 0.2922 |
